# Accessibility and quality of medical care for patients with chronic noncommunicable diseases during COVID-19 pandemic

**DOI:** 10.1038/s41533-023-00328-9

**Published:** 2023-03-31

**Authors:** Andrey Reshetnikov, Irina Frolova, Olga Abaeva, Nadezhda Prisyazhnaya, Tatyana Romanova, Sergey Romanov, Konstantin Sobolev

**Affiliations:** 1 Institute of Social Sciences, Federal State Autonomous Educational Institution of Higher Education I.M. Sechenov First Moscow State Medical University of the Ministry of Health of the Russian Federation (Sechenov University), Moscow, Russian Federation; 2 Volga district medical center under Federal medical-biology agency, N. Novgorod, Russian Federation; 3 State Budgetary Institution of Health of the Moscow Region “Moscow Regional Research Clinical Institute named after M.F. Vladimirsky” (GBIH MR MRRCI named after M.F. Vladimirsky), Moscow, Russian Federation

**Keywords:** Health care, Respiratory tract diseases

## Abstract

The purpose of this study is to conduct a comparative analysis of the impact of the accessibility and quality of medical care provided to patients with chronic noncommunicable diseases (CNCDs) during COVID-19 pandemic on the course and outcome of COVID-19 infection. The study included 132 patients hospitalized with a diagnosis of COVID-19 and having one or more concomitant CNCDs. The patients were divided into two groups based on the quality of the initial CNCD therapy they received. Group 1 involved 58 patients (42%) who received treatment according to clinical guidelines and had a compensated CNCD. Group 2 consisted of 76 patients (58%) who received treatment that was not in line with modern clinical guidelines and/or had a decompensated CNCD. All ‘red zone’ hospitalized patients were surveyed. In particular, they were asked questions related to the quality and accessibility of medical care during COVID-19 pandemic and their satisfaction with the medical care received during the pandemic. Reduced access to medical care (the failure to have the therapy received timely evaluated and adjusted) during COVID-19 pandemic affects the quality of the therapy received by patients with CNCDs. Generally, an unfavorable course and outcome of COVID-19 infection are typical for patients receiving a non-optimal CNCD therapy as compared to patients receiving a therapy that meets current clinical guidelines.

## Introduction

COVID-19 pandemic is one of the world’s biggest challenges. It is responsible for a crisis in the healthcare and economic sectors. The situation is further aggravated by the fact that COVID-19 pandemic has overlapped with the already striking CNCD epidemic, which has accounted for 72% of deaths worldwide^1^. The presence of one or more CNCDs in a patient infected with SARS-COV-2, including cardiovascular diseases, chronic respiratory diseases (such as chronic obstructive pulmonary disease), diabetes, and cancer, predetermine the unfavorable course and prognosis of COVID-19 infection^2–6^.

This mutual burden of CNCD and COVID-19 infection is owing to the possible association between multiple diseases, such as hypertension, diabetes, respiratory and cardiovascular diseases, and COVID-19 pathogenesis. Chronic diseases share a number of features with infectious diseases, such as a pro-inflammatory state and weakened innate immune response^7^. A single pathway for the activation of the renin-angiotensin-aldosterone system (RAAS) and penetration of SARS-COV-2 into the host cell is mediated by the ACE2 receptor. Infection causes cardiovascular damage^8^. The development of diabetes is associated with the accumulation of activated innate immune cells, leading to the release of inflammatory mediators, which contribute to systemic insulin resistance and β-cells damage^9^. Metabolic disorders themselves can suppress immune function due to the impaired function of macrophages and lymphocytes.

Thus, the current situation, which presents a combination of CNCD epidemic and COVID-19 pandemic, is complex. On the one hand, implementation of the measures aimed at prevention or reduction of the number of new cases of SARS-CoV-2 infection in the form of self-isolation reduce the risk of infection. On the other hand, these measures cause a lot of problems in patients with CNCDs, resulting in limited or no access to treatment^10^, restricted access to pharmacies and pharmaceuticals, cancellation of scheduled doctor’s appointments, and planned surgery delays^11^. Chronically ill patients may experience additional fears and anxieties related to their illnesses, care, and therapy^12^.

Spending more time indoors leads to an increase in the impact of some adverse factors, including unhealthy diet, alcohol consumption, and stress. These are the risk factors for CNCDs^13,14^.

The importance of CNCD prevention and treatment during COVID-19 pandemic is underlined by the fact that the true CNCD rate is underestimated. This is because many diseases are not diagnosed on time^15,16^ and are not effectively treated^17^.

Various sociological surveys have been conducted during the pandemic, including remote ones (telephone interviews), to assess the impact of COVID-19 pandemic on the health status of patients with CNCDs^18^. The surveys undertaken have identified that patients with CNCDs have an increased need for medical care and information regarding different risks and opportunities for preventing both CNCD complications and COVID-19 infection. Economic and social status is importance. An American study^19^, which evaluated the changes in the structure of medical care associated with the social restrictions policy (SIP-shelter-in-place), found that self-isolation strategy led to a 10% reduction in the number of doctor visits and a 53% increase in the use of telemedicine services. However, telemedicine compensated for the doctor visits only in half of cases^20^. It was difficult to assess the treatment quality since the majority of the respondents (40.4%) visited doctors for non-urgent reasons (administrative issues: prescription, referral to a specialist, sick leave) and only 12.1% of the respondents had teleconsultations related to the treatment of their chronic diseases^21^.

Thus, most studies were conducted on patients with CNCDs who did not have COVID-19 infection, often remotely, regardless of the so-called COVID status. There are insufficient data on the satisfaction of CNCD patients hospitalized with COVID-19 pneumonia with the medical care they received. In connection with the above, several research questions have been identified. Firstly, the researchers are willing to find out whether patient satisfaction with the medical care received during the pandemic is synonymous with high medical care quality. Secondly, this paper will investigate whether COVID-19 infection is milder in patients receiving high-quality treatment for CNCDs (in accordance with modern clinical recommendations) as compared to patients receiving poor-quality treatment or suffering from decompensated CNCDs. Lastly, the study will predict the rate of hospitalizations due to decompensated medical conditions after COVID-19 pandemic subsides.

Evaluation of the accessibility of medical care and its effectiveness can be useful for patients, doctors, researchers, and healthcare providers. Thus, the present study is important in light of the fact that COVID-19 impact is likely to linger for years.

## Methods

### Research design and sample

Among 208 patients admitted to the Federal Budgetary Healthcare Institution Privolzhye District Medical Center of the Federal Medical and Biological Agency (FBHI PDMC of the FMBA) of Russia, Temporary Infectious Diseases Hospital Clinical Hospital No. 2, Nizhny Novgorod, during the period from 03 January 2021 to 03 March 2021, 158 patients (76%) with one or more chronic CNCDs were selected. Of them, 132 patients were included in the retrospective analysis. The study enrolled both men and women aged over 18 who were diagnosed with COVID-19, had one or more concomitant CNCDs, and received treatment in the in-hospital setting. All participants gave written informed consent to participate in the research. The study excluded patients who were aged less than 18 years at the time of hospitalization, and those who received outpatient treatment and had no concomitant CNCDs. In addition, enrollment did not occur for patients who received no prehospital CNCD therapy or failed to complete the questionnaire due to the severity of their condition. All patients underwent examination, which involved measuring blood pressure (BP), heart rate (HR), and laboratory parameters (low-density lipoprotein cholesterol (LDL-C), glucose, and hemoglobin). Based on the results of the examination, instrumental data, and modern clinical recommendations, two study groups were formed. Group 1 involved 58 patients (42%) who received treatment according to clinical guidelines and had a compensated CNCD. Group 2 consisted of 76 patients (58%) who received treatment that was not in line with modern clinical guidelines and/or had a decompensated CNCD. The quality of prehospital CNCD therapy was assessed individually for each patient. Upon admission to the hospital, all “red zone” patients were surveyed. In particular, they were asked questions related to the quality and accessibility of medical care during COVID-19 pandemic and their satisfaction with the medical care received during the pandemic (the questionnaire administered is attached). The answers given by the study groups were compared.

Demographics (age, gender, and race) and clinical data (medical history, medications taken at admission, signs, and symptoms at admission, and physical examination at admission) were collected. Clinical course and complications of COVID-19 during hospital stay were obtained from CRFs. A standard data collection form was used at admission and discharge. A sociological tool was applied, with each patient interviewed individually.

The primary endpoint was in-hospital deaths. The secondary endpoints included the need for various types of oxygen therapy, development of cytokine storm, development of COVID-19 complications, duration of fever, length of ICU stay, and length of hospital stay.

### Statistical processing

Statistical processing was carried out using Statistica 10.0 (StatSoft, USA). Categorical variables are presented as *n* (%). Continuous variables are described by medians with lower and upper quartiles. Intergroup differences were checked using the Student’s *t* test for normally distributed data and Mann–Whitney *U* test for non-normally distributed data. The proportions were compared using the chi-square test or Fisher’s exact test, where appropriate.

### Ethics

All participants provided written informed consent to take part in the study. The study was approved by the Ethics Committee at the FBHI PDMC of the FMBA of Russia, Nizhny Novgorod. An electronic case report form (CRF) was completed for each patient. All study documents were prepared only in electronic format. An informed consent for the use of these medical documents for study purposes was given by all patients.

### Reporting summary

Further information on research design is available in the Nature Research Reporting Summary linked to this article.

## Results

### Analysis of therapy

The median and interquartile age of the study subjects equaled 64.0 [53.0; 72.0] years. Considering gender distribution, 45% (*n* = 59) of the study participants were men and 55% (*n* = 73) were women.

AH was the most common CNCDs. It was observed in 90% of cases, with similar rates reported for the group receiving optimal (group 1) and non-optimal (group 2) therapies (89 and 93%, respectively, as shown in Table 1). Obesity was detected in 46% of study participants. Interestingly, patients receiving non-optimal therapy had obesity in 55% cases, and this value is significantly higher than the one confirmed for the group receiving optimal therapy (34% at *p* < 0.05). T2DM was diagnosed in 27%, CHD—in 15%, CHF—in 20% of patients. Oncology was found in 12% of patients. AF was detected in 8%, MI—in 8%, a history of stroke—in 10%, CKD—in 6%, COPD—in 3%, and bronchial asthma—in 5% of cases. As to the diseases listed above, there were no statistically significant intergroup differences revealed (Table 1).

**Table 1:** Study groups by demography, medical history, and clinical picture.

Parameter	Total population (*n* = 132)	Non-optimal CNCD therapy (*n* = 76)	Optimal CNCD therapy (*n* = 58)	*p* value
Age, years (Me [Q25; Q75])	64 [53; 72]	67 [59; 75]	64 [57; 72]	0.19
Men, *n* (%)	59 (45)	36 (49)	23 (40)	0.39
AH, *n* (%)	120 (90)	66 (89)	54 (93)	0.63
Smoking, *n* (%)	6 (5)	4 (5)	2 (3)	0.90
Obesity (BMI ≥ 30 kg/m^2^), *n* (%)	61 (46)	41 (55)	20 (34)	(<) 0.05
AF, *n* (%)	11 (8)	6 (8)	5 (9)	0.83
CHD (including history of MI), *n* (%)	21 (15)	14 (19)	7 (12)	0.40
History of MI, *n* (%)	10 (8)	7 (9)	3 (5)	0.55
CHF, *n* (%)	26 (20)	16 (22)	10 (17)	0.68
History of ACVA, *n* (%)	13 (10)	10 (14)	3 (5)	0.19
T2DM, *n* (%)	36 (27)	26 (35)	10 (17)	0.03
CKD, *n* (%)	8 (6)	5 (8)	3 (5)	1.00
Chronic bronchitis, *n* (%)	4 (3)	2 (3)	2 (3)	1.00
Bronchial asthma, *n* (%)	8 (5)	2 (3)	6 (10)	(>) 0.05
History of cancer, *n* (%)	16 (12)	12 (16)	4 (7)	0.17
1 CD	33 (25)	8 (10)	25 (43)	0.0005
2 or 3 CDs	67 (51)	44 (59)	23 (40)	0.03
4 and more CDs	28 (21)	18 (24)	10 (17)	0.44

*AH* arterial hypertension, *BMI* body mass index, *CHD* coronary heart disease, *MI* myocardial infarction, *ACVA* acute cerebrovascular accident, *T2DM* type 2 diabetes mellitus, *CHF* chronic heart failure, *AF* atrial fibrillation, *CKD* chronic kidney disease, *CNCDs* chronic noncommunicable diseases, *COPD* chronic obstructive pulmonary disease.

Highlighted are the indicators calculated at *p* < 0.05. They allow better visualization of the tabular data.

One CD was present in 25% of patients. Meanwhile, it is worth noting that the majority of patients receiving an optimal therapy had one CD (*p* = 0.0005). Two or three CDs were observed in 51% of patients, and there were some differences identified: 59% in the optimal therapy group and 40% in the optimal therapy group (*p* = 0.03). Four concomitant diseases and more were revealed in 20% of patients, with no statistically significant intergroup differences identified.

Half of the surveyed patients (50%) reported health deterioration during the pandemic. Group 1 complained of worsening health in 40% of cases (*n* = 23), and group 2—in 58% of cases (*n* = 43, *p* = 0.035, as shown in Table 2).

**Table 2:** Study groups by the quality of treatment.

Parameter	The number of surveyed patients	Total population (*n* = 132)	Non-optimal CNCD therapy (*n* = 76)	Optimal CNCD therapy (*n* = 58)	*p* value
The patient reported health deterioration during the pandemic, *n* (%)	132	66 (50)	43 (58)	23 (40)	0.035
The patient required therapy adjustment during the pandemic, *n* (%)	132	62 (46)	43 (58)	19 (32)	0.0065
Last therapy adjustment, time interval (Me [Q25; Q75])		4 [3; 4]	4 [3; 4]	4 [3; 4]	0.69
Therapy adjusted less than 1 month ago, *n* (%)	128	11 (9)	7 (10)	4 (7)	1.00
Therapy adjusted 1–6 months ago, *n* (%)	128	23 (18)	14 (19)	9 (16)	1.00
Therapy adjusted 6–12 months ago, *n* (%)	128	27 (21)	18 (25)	9 (16)	0.66
Therapy adjusted more than 1 year ago, *n* (%)	128	67 (52)	34 (47)	33 (60)	0.61
Last doctor visit for concomitant diseases, time interval (Me [Q25; Q75])		4 [2; 4]	4 [3; 4]	4 [2; 4]	0.72
The patient visited his/her doctor <1 month ago, *n* (%)	128	10 (8)	7 (10)	3 (5)	0.53
The patient visited his/her doctor 1–6 months ago, *n* (%)	128	26 (20)	14 (19)	12 (21)	0.38
The patient visited his/her doctor 6–12 months ago, *n* (%)	128	27 (21)	19 (26)	8 (14)	0.38
The patient visited his/her doctor more than 1 year ago, *n* (%)	128	65 (51)	33 (45)	32 (58)	0.61
The patient required inpatient care during the pandemic, *n* (%)	132	21 (16)	14 (19)	7 (12)	0.40
The patient complaint of reduced access to medical care during the pandemic, *n* (%)	132	55 (42)	36 (49)	19 (33)	0.09
The patient did not receive medical care due to unavailability of the needed healthcare professional at the medical institution, *n* (%)	55	19 (35)	13 (36)	6 (32)	1.00
The patient did not receive medical care due to the failure to make an appointment, *n* (%)	55	21 (38)	15 (42)	6 (32)	0.27
The patient did not receive medical care due to no spare time in his agenda, *n* (%)	55	2 (4)	1 (3)	1 (5)	1.00
The patient did not receive medical care due to the medical institution reprofiling for COVID-19 infection, *n* (%)	55	13 (24)	7 (19)	6 (32)	0.31
The patient considers his/her treatment sufficient, *n* (%)	126	102 (78)	57 (78)	45 (83)	0.61
The patient continued to administer all drugs prescribed during the pandemic, *n* (%)	126	111 (88)	61 (85)	50 (92)	0.28
The preceding therapy was discontinued due to the fear of going to the pharmacy because of the risk of COVID-19 infection, *n* (%)	15	2 (18)	0 (0)	2 (50)	0.11
The preceding therapy was discontinued due to unavailability of a pharmacy nearby, *n* (%)	15	2 (18)	2 (29)	0 (0)	0.49
The preceding therapy was discontinued due to the rapid increase in the prices for pharmaceuticals, *n* (%)	15	2 (18)	1 (14)	1 (25)	1.00
The preceding therapy was discontinued due to the deteriorating financial status of the family, *n* (%)	15	5 (45)	4 (57)	1 (25)	0.55

Among patients receiving prehospital therapy, almost half of the responders (46%, *n* = 62) required adjustment of their basic therapy. Such a need was two times more common among group 2 patients (58%, *n* = 43) as compared to group 1 patients (33%, *n* = 19, *p* = 0.0065).

### Analysis of the actual period of therapy

During an analysis of the actual period of therapy adjustment made for all treated CNCD patients, it turned out that half of all patients in the two groups (52%, *n* = 67) had their therapy adjusted before the start of the pandemic, i.e., more than a year before, 18% (*n* = 23) of patients—within the previous 6 months, 21% (*n* = 27)—6–12 months before, and 4% (*n* = 6) did not require therapy adjustment at all. The period of therapy adjustment in both study groups was 4 [3; 4] months, with no statistically significant intergroup difference demonstrated (*p* = 0.69). Similar results were obtained for the last doctor’s appointment (Table 2).

The need for inpatient care was recognized by 16% of the respondents (*n* = 21). There were 12% (*n* = 7) of patients requiring care in the inpatient setting in group 1 and 19% (*n* = 14)—in group 2 (*p* = 0.342).

Subjectively, reduced access to medical care was noted by 42% of the respondents (*n* = 55). This problem was experienced by 33% (*n* = 19) of group 1 patients and 49% (*n* = 36) of group 2 patients (*p* = 0.066). In 73% of cases (*n* = 40), the reasons for not receiving medical care were the unavailability of the healthcare professional needed and failure to make an appointment. Only 24% (*n* = 13) of patients explained their limited access to healthcare services by medical institutions reprofiling.

The proportion of patients who considered their treatment sufficient was quite high (78%, *n* = 102). It was similar in both groups, as this opinion shared 83% of patients (*n* = 45) in group 1 and 78% of patients (*n* = 57) in group 2 (*p* = 0.61).

Remarkably, only 12% (*n* = 15) of patients receiving treatment decided to discontinue their therapy during COVID-19 pandemic on their own. These were mainly group 2 patients (11 out of 15). There were 7% (*n* = 4) of patients who stopped the prescribed therapy in group 1 and 15% (*n* = 11)—in group 2 (*p* = 0.263).

The reasons for therapy discontinuation included worsening financial status and an increase in treatment costs. These answers were given by 66% of the respondents (*n* = 7). There were only two individuals who had a fear of going to the pharmacy because of the risk of contracting the virus (*p* = 0.268, Table 2).

The therapies received by the patients at the pre-hospitalization stage were analyzed. This analysis revealed that group 1 received angiotensin-converting enzyme inhibitors (ACE inhibitors), beta-blockers (BB), calcium channel blockers, and statins more often as compared to group 2 (Table 3).

**Table 3:** Study groups by the preceding CNCD therapy.

Parameter, *n* (%)	Total population (*n* = 132)	Non-optimal CNCD therapy (*n* = 76)	Optimal CNCD therapy (*n* = 58)	*p* value
ACE inhibitor therapy	34 (22)	16 (16)	18 (31)	0.04
Angiotensin receptor blocker (ARB) therapy	55 (35)	32 (32)	23 (40)	0.39
Beta-blocker therapy	54 (34)	27 (27)	27 (47)	0.02
AK therapy	46 (29)	21 (21)	25 (43)	0.004
Diuretic therapy	37 (23)	21 (21)	16 (28)	0.44
Statin therapy	31 (20)	14 (14)	17 (29)	0.02
Anticoagulant therapy	26 (16)	12 (12)	14 (24)	0.07
Antiplatelet therapy	28 (18)	16 (16)	12 (21)	0.52
T2DM hypoglycemic therapy	33 (21)	24 (24)	9 (16)	0.23
Bronchodilator therapy	5 (3)	2 (2)	3 (5)	0.36

*T2DM* type 2 diabetes mellitus.

### Analysis of clinical characteristics of COVID-19 course

The clinical characteristics of COVID-19 course were compared. This comparison confirmed that the rates of certain complications, such as cytokine storm, acute kidney injury, sepsis, and infective toxic shock, were higher among group 2 than among group 1 patients (Table 4).

**Table 4:** Study groups by the course and outcome of COVID-19.

Parameter, *n* (%)	Total population (*n* = 132)	Non-optimal CNCD therapy (*n* = 76)	Optimal CNCD therapy (*n* = 58)	*p* value
Death, *n* (%)	15 (11)	14 (19)	1 (2)	0.005
Acute coronary syndrome, *n* (%)	3 (2)	3 (3)	0 (0)	0.30
Acute cerebrovascular accident, *n* (%)	1 (1)	1 (1)	0 (0)	1.00
Cytokine storm, *n* (%)	50 (38)	34 (46)	16 (28)	0.04
Acute kidney injury (KDIGO criteria), *n* (%)	28 (18)	18 (24)	5 (9)	0.03
Sepsis, infective toxic shock, *n* (%)	12 (9)	11 (15)	1 (2)	0.006
No oxygen therapy, *n* (%)	30 (23)	13 (18)	17 (29)	0.17
Humidified oxygen, *n* (%)	79 (60)	41 (55)	38 (65)	0.23
High-flow oxygenation, *n* (%)	12 (9)	10 (14)	2 (3)	0.045
Non-invasive ALV, *n* (%)	2 (1)	1 (1)	1 (2)	1.00
ALV, *n* (%)	9 (7)	9 (12)	0 (0)	0.005
Fever duration, days (Me [Q25; Q75])	9 [7; 11]	10 [7; 12]	9 [7; 10]	0.08
ICU stay, days (Me [Q25; Q75])	0 [0; 0]	0 [0; 6]	0 [0; 0]	<0.001
Hospital stay, days (Me [Q25; Q75])	9 [7; 13]	10 [8; 14]	8 [7; 11]	0.002

*ALV* artificial lung ventilation.

The intensity of oxygen therapy was higher in group 2 as compared to group 1. The requirement for high-flow oxygenation and mechanical ventilation was higher among group 2 than among group 1 patients (Table 4).

Referring to the primary endpoint, in-hospital mortality was significantly higher among group 2 patients as compared to group 1 patients (21% versus 2%, *p* = 0.005, as shown in Table 4). As to the secondary endpoints, group 2 patients (non-optimal therapy) were found to have a significantly longer period of ICU stay as compared to group 1 patients (optimal therapy) (0 [0; 6] versus 0 [0; 0] days, *p* < 0.001). Moreover, group 2 patients had a longer hospital stay than group 1 patients (10 [8; 14] versus 8 [7; 11] days, *p* = 0.002, as shown in Table 4).

## Discussion

The majority of patients admitted for inpatient treatment had one or more CNCDs (76% of all hospitalized patients, *n* = 158). This is due to the fact that COVID-19 infection has a more unfavorable course in patients with cardiovascular, oncological, and bronchopulmonary diseases, diabetes, and obesity^22,23^. Only 42% (*n* = 58) of patients received quality CNCD treatment. The remaining 58% received treatment that did not meet current clinical guidelines and had decompensated CNCDs. Despite the worsening health status and the need to adjust therapy during the pandemic in half of the study participants, 52% of them had their therapy adjusted even before the start of the pandemic. At the same time, 42% of the respondents reported receiving worse medical care during the epidemic. This is because the rather low quality of medical care received for CNCDs before the pandemic worsened even more due to subjective and objective factors associated with both quarantine measures in the form of self-isolation, which had an impact on behavioral risk factors, psychosomatic status, and clinical course of the disease, and the healthcare system burden associated with the reprofiling of medical institutions. The volume of both planned and emergency care offered to patients during the pandemic was reduced. Thus, during the period of another increase in the incidence of COVID-19 in the United States, Californian researchers confirmed a decrease in the frequency of hospitalizations due to acute coronary syndrome (ACS)^24^. Similar data were obtained in Italy, where an increase in the incidence of complications and mortality was documented^25^. The INCAPS COVID Investigators Group international study, which included 909 hospitals, revealed a decreased frequency of invasive and non-invasive examinations during COVID-19 pandemic^26^.

Meanwhile, the subjective assessment of the therapy quality was quite high. Indeed, 78% of the respondents considered their treatment sufficient, and 88% of the study participants declared their adherence to the therapy prescribed.

The questionnaire administered in the present study revealed statistically significant intergroup differences only in terms of the condition worsening during the pandemic and the need for treatment adjustment. In addition, there were significant differences in the course and outcomes of COVID-19 infection. Therefore, patients’ favorable subjective assessment of the quality of the treatment received does not mean that the therapy was complete, sufficient, and adequate. Such an assessment requires an integrated approach incorporating objective criteria based on clinical recommendations and the use of various scales. During the pandemic, when there are fewer opportunities for a patient with a CNCD to receive medical care (and these are people who have the most unfavorable prognosis for COVID-19 infection), it is very important to actively monitor such patients, objectively evaluate the therapy they receive, and timely adjust it at the polyclinic level. Therefore, the main method of preventing severe complications of COVID-19 infection in patients with CNCDs is dispensary observation of such patients, despite all the challenges that primary healthcare is facing during the pandemic.

In patients with chronic noncommunicable diseases hospitalized with COVID-19, cardiovascular diseases, obesity, and T2DM predominate. The data obtained through the questionnaire indicate a decrease in the accessibility of medical care during the pandemic. At the same time, the quality of the therapy received and the levels of adherence to the CNCD treatment are assessed by the patients as quite high. Nevertheless, these data are subjective and do not reflect the true quality of the therapy received. Importantly, this issue should be considered during COVID-19 treatment, since patients receiving a basic CNCD therapy that is compliant with current clinical guidelines before their admission to the infectious disease unit has a more favorable course of COVID-19 infection at the hospital stage and a lower level of in-hospital mortality as compared to patients receiving non-optimal therapy. Prospects for further research are based on the possibility of further study of the problem of treatment of chronic noncommunicable diseases, and search for new effective methods and treatment approaches not only in the conditions of a pandemic, but also in the modern world in general. The branch of pharmacology is promising, in particular, the development of new medicinal products with fewer adverse reactions and greater prolonged action in the treatment of infectious diseases. Therefore, the developed methodology can be used in other areas, in particular, medicine, pharmacology, and psychology (for example, to study the impact of infectious diseases on the development of mental disorders in patients in the context of the COVID-19 pandemic).

## Supplementary information

Supplementary Information

Reporting Summary

## Data Availability

Data will be available on request.
