# Supplementary material for: Accessibility and quality of medical care for patients with chronic noncommunicable diseases during COVID-19 pandemic
Source: NPJ Prim Care Respir Med. 2023 Mar 31;33:14. doi: 10.1038/s41533-023-00328-9 (PMC10063946; doi:10.1038/s41533-023-00328-9)
Supplement: Supplementary file 1 — Supplementary Information [file 41533_2023_328_MOESM1_ESM.pdf]

Supplementary Information

Questionnaire:

Full name	
Diagnoses:	
Drugs administered (tradenames) and doses:	
Have you noticed a health decline during the pandemic?	Yes<br>No
Have you required therapy adjustment during the pandemic?	Yes<br>No
When have you had your therapy last adjusted?	1. < 1 month ago<br>2. 1-6 months ago<br>3. 6-12 months ago<br>4. > 1 year ago
When did you last visit the doctor to talk about your diseases?	1. < 1 month ago<br>2. 1-6 months ago<br>3. 6-12 months ago<br>4. > 1 year ago
Did you require in-hospital care?	Yes<br>No
It was more challenging to apply for medical care during the pandemic?	Yes<br>No

Why you did not receive medical care?	1. Medical institution did not employ a healthcare professional I needed<br>2. There were no appointments available<br>3. I did not have time to go to the doctor<br>4. My medical institution was reprofiled for COVID-19
In your opinion, was your treatment sufficient?	Yes<br>No
Have you continued to take all of your medications during the pandemic?	Yes<br>No
Why did you discontinue your previous therapy?	1. Fear of going to the pharmacy because of the risk of contracting COVID-19<br>2. No pharmacy within walking distance<br>3. The prices for pharmaceuticals increase too fast<br>4. The financial status of my family has deteriorated
