# Supplementary material for: Accessibility and quality of medical care for patients with chronic noncommunicable diseases during COVID-19 pandemic
Source: NPJ Prim Care Respir Med. 2023 Mar 31;33:14. doi: 10.1038/s41533-023-00328-9 (PMC10063946; doi:10.1038/s41533-023-00328-9)
Supplement: Supplementary file 2 — Reporting Summary [file 41533_2023_328_MOESM2_ESM.pdf]

nature portfolio

Corresponding author(s): Andrey Reshetnikov

Last updated by author(s): Nov 3, 2022

Reporting Summary

Nature Portfolio wishes to improve the reproducibility of the work that we publish. This form provides structure for consistency and transparency in reporting. For further information on Nature Portfolio policies, see our Editorial Policies and the Editorial Policy Checklist.

Statistics

For all statistical analyses, confirm that the following items are present in the figure legend, table legend, main text, or Methods section.

n/a	Confirmed	
☐	☒	The exact sample size (*n*) for each experimental group/condition, given as a discrete number and unit of measurement
☐	☒	A statement on whether measurements were taken from distinct samples or whether the same sample was measured repeatedly
☐	☒	The statistical test(s) used AND whether they are one- or two-sided<br>*Only common tests should be described solely by name; describe more complex techniques in the Methods section.*
☐	☒	A description of all covariates tested
☐	☒	A description of any assumptions or corrections, such as tests of normality and adjustment for multiple comparisons
☐	☒	A full description of the statistical parameters including central tendency (e.g. means) or other basic estimates (e.g. regression coefficient) AND variation (e.g. standard deviation) or associated estimates of uncertainty (e.g. confidence intervals)
☐	☒	For null hypothesis testing, the test statistic (e.g. *F*, *t*, *r*) with confidence intervals, effect sizes, degrees of freedom and *P* value noted<br>*Give P values as exact values whenever suitable.*
☐	☒	For Bayesian analysis, information on the choice of priors and Markov chain Monte Carlo settings
☐	☒	For hierarchical and complex designs, identification of the appropriate level for tests and full reporting of outcomes
☐	☒	Estimates of effect sizes (e.g. Cohen's *d*, Pearson's *r*), indicating how they were calculated

Our web collection on statistics for biologists contains articles on many of the points above.

Software and code

Policy information about availability of computer code

Data collection	Statistical processing was carried out using Statistica 10.0 (StatSoft, USA).
Data analysis	Statistical processing was carried out using Statistica 10.0 (StatSoft, USA).

For manuscripts utilizing custom algorithms or software that are central to the research but not yet described in published literature, software must be made available to editors and reviewers. We strongly encourage code deposition in a community repository (e.g. GitHub). See the Nature Portfolio guidelines for submitting code & software for further information.

Data

Policy information about availability of data

All manuscripts must include a data availability statement. This statement should provide the following information, where applicable:

- Accession codes, unique identifiers, or web links for publicly available datasets
- A description of any restrictions on data availability
- For clinical datasets or third party data, please ensure that the statement adheres to our policy

All data generated or analysed during this study are included in this published article.

Human research participants

Policy information about studies involving human research participants and Sex and Gender in Research.

Reporting on sex and gender	The study enrolled both men and women aged over 18 who were diagnosed with COVID-19, had one or more concomitant CNCDs and received treatment in the in-hospital setting.
Population characteristics	The study enrolled both men and women aged over 18 who were diagnosed with COVID-19, had one or more concomitant CNCDs and received treatment in the in-hospital setting.
Recruitment	Among 208 patients admitted to the Federal Budgetary Healthcare Institution Privolzhye District Medical Center of the Federal Medical and Biological Agency (FBHI PDMC of the FMBA) of Russia, Temporary Infectious Diseases Hospital Clinical Hospital No. 2, Nizhny Novgorod, during the period from January 03, 2021 to March 03, 2021, 158 patients (76%) with one or more chronic CNCDs were selected. Of them, 132 patients were included in the retrospective analysis
Ethics oversight	The research was conducted ethically in accordance with the World Medical Association Declaration of Helsinki. The research was approved by the local ethics committees of Sechenov University.

Note that full information on the approval of the study protocol must also be provided in the manuscript.

Field-specific reporting

Please select the one below that is the best fit for your research. If you are not sure, read the appropriate sections before making your selection.

☒ Life sciences ☐ Behavioural & social sciences ☐ Ecological, evolutionary & environmental sciences

For a reference copy of the document with all sections, see nature.com/documents/nr-reporting-summary-flat.pdf

Life sciences study design

All studies must disclose on these points even when the disclosure is negative.

Sample size	Among 208 patients admitted to the Federal Budgetary Healthcare Institution Privolzhye District Medical Center of the Federal Medical and Biological Agency (FBHI PDMC of the FMBA) of Russia, Temporary Infectious Diseases Hospital Clinical Hospital No. 2, Nizhny Novgorod, during the period from January 03, 2021 to March 03, 2021, 158 patients (76%) with one or more chronic CNCDs were selected. Of them, 132 patients were included in the retrospective analysis.
Data exclusions	The study excluded patients who were aged less than 18 years at the time of hospitalization, those who received outpatient treatment and had no concomitant CNCDs. In addition, enrolment did not occur for patients who received no prehospital CNCD therapy or failed to complete the questionnaire due to the severity of their condition.
Replication	All patients underwent examination, which involved measuring blood pressure (BP), heart rate (HR), and laboratory parameters (low-density lipoprotein cholesterol (LDL-C), glucose, and hemoglobin).
Randomization	Group 1 involved 58 patients (42%) who received treatment according to clinical guidelines and had a compensated CNCD. Group 2 consisted of 76 patients (58%) who received treatment that was not in line with modern clinical guidelines and/or had a decompensated CNCD.
Blinding	An informed consent for the use of these medical documents for the study purposes was given by all patients. The investigators wew blinded.

Reporting for specific materials, systems and methods

We require information from authors about some types of materials, experimental systems and methods used in many studies. Here, indicate whether each material, system or method listed is relevant to your study. If you are not sure if a list item applies to your research, read the appropriate section before selecting a response.

Materials & experimental systems

n/a	Involved in the study
☒	☐ Antibodies
☒	☐ Eukaryotic cell lines
☒	☐ Palaeontology and archaeology
☒	☐ Animals and other organisms
☒	☐ Clinical data
☒	☐ Dual use research of concern

Methods

n/a	Involved in the study
☒	☐ ChIP-seq
☒	☐ Flow cytometry
☒	☐ MRI-based neuroimaging
